# Supplementary material for: Reconstruction of the tumor spatial microenvironment along the malignant-boundary-nonmalignant axis
Source: Nat Commun. 2023 Feb 20;14:933. doi: 10.1038/s41467-023-36560-7 (PMC9941488; doi:10.1038/s41467-023-36560-7)
Supplement: Supplementary file 1 — Supplementary Information [file 41467_2023_36560_MOESM1_ESM.pdf]

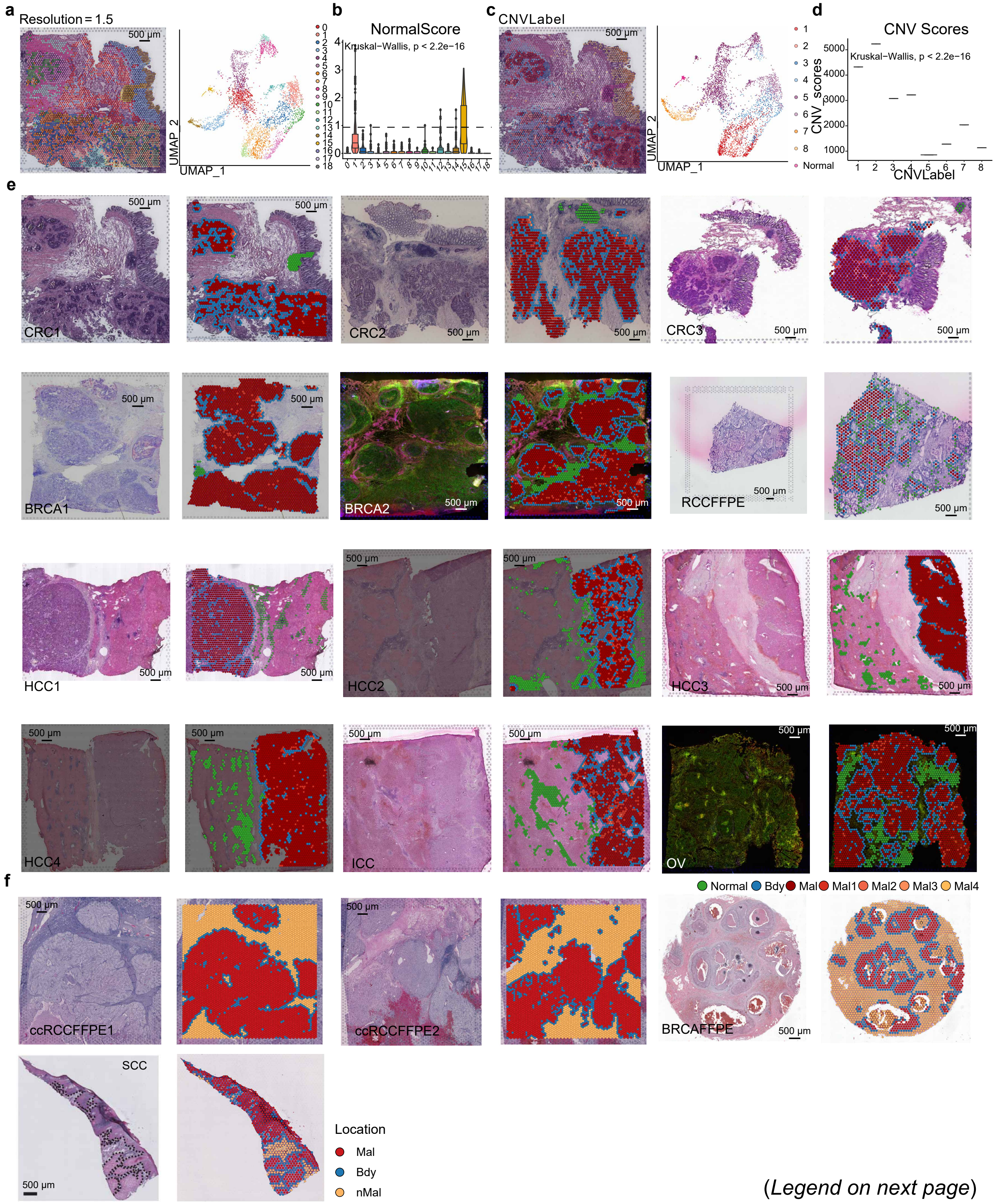

(*Legend on next page*)

Supplementary Fig. 1 Delineating the tumor boundary. Related to Figure 2. (**a**) Spatial dim plots (left) and UMAP projection (right) showing the KNN clustering of spots based morphological adjusted ST matrix. Colors represent the cluster labels. (**b**) Box plots showing the distribution of normal score calculated by immune signature (see Methods) among different KNN clusters (spots of each cluster: n_{c0} = 620, n_{c1} = 457, n_{c2} = 357, n_{c3} = 332, n_{c4} = 321, n_{c5} = 307, n_{c6} = 300, n_{c7} = 299, n_{c8} = 297, n_{c9} = 250, n_{c10} = 179, n_{c11} = 165, n_{c12} = 118, n_{c13} = 120, n_{c14} = 117, n_{c15} = 79, n_{c16} = 78, n_{c17} = 42, n_{c18} = 13). The boxes show the median ± 1 quartile, with the whiskers extending from the hinge to the smallest or largest value within 1.5 × the IQR from the box boundaries. (**c**) Spatial dim plots (left) and UMAP projection (right) showing the clustering of spots based on CNV matrix. (**d**) The distribution of CNV score in clusters defined in (**c**). (**e**) Spatial location showing tumor layers defined by extrapolation from core spots of malignant spots according to the hexagonal system, with different depths in red representing the extrapolated malignant spots for each layer, the final defined boundary spots (blue) and the locations of tissue (green) used as normal reference for infercnv calculations. (**f**) Tissue slides were annotated by malignant spots (Mal, red), boundary spots (Bdy, blue), and non-malignant spots (nMal, orange), including ccRCC (FFPE samples, n = 2), breast cancer (FFPE sample, n = 1), and squamous cell carcinoma (SCC, n=1). Scale bar, 500 μm in **a**, **c**, **e-f**. UMAP, Uniform Manifold Approximation and Projection; FFPE, formalin-fixed, paraffin-embedded. Source data are provided as Source Data Supplementary Figure 1e-f.

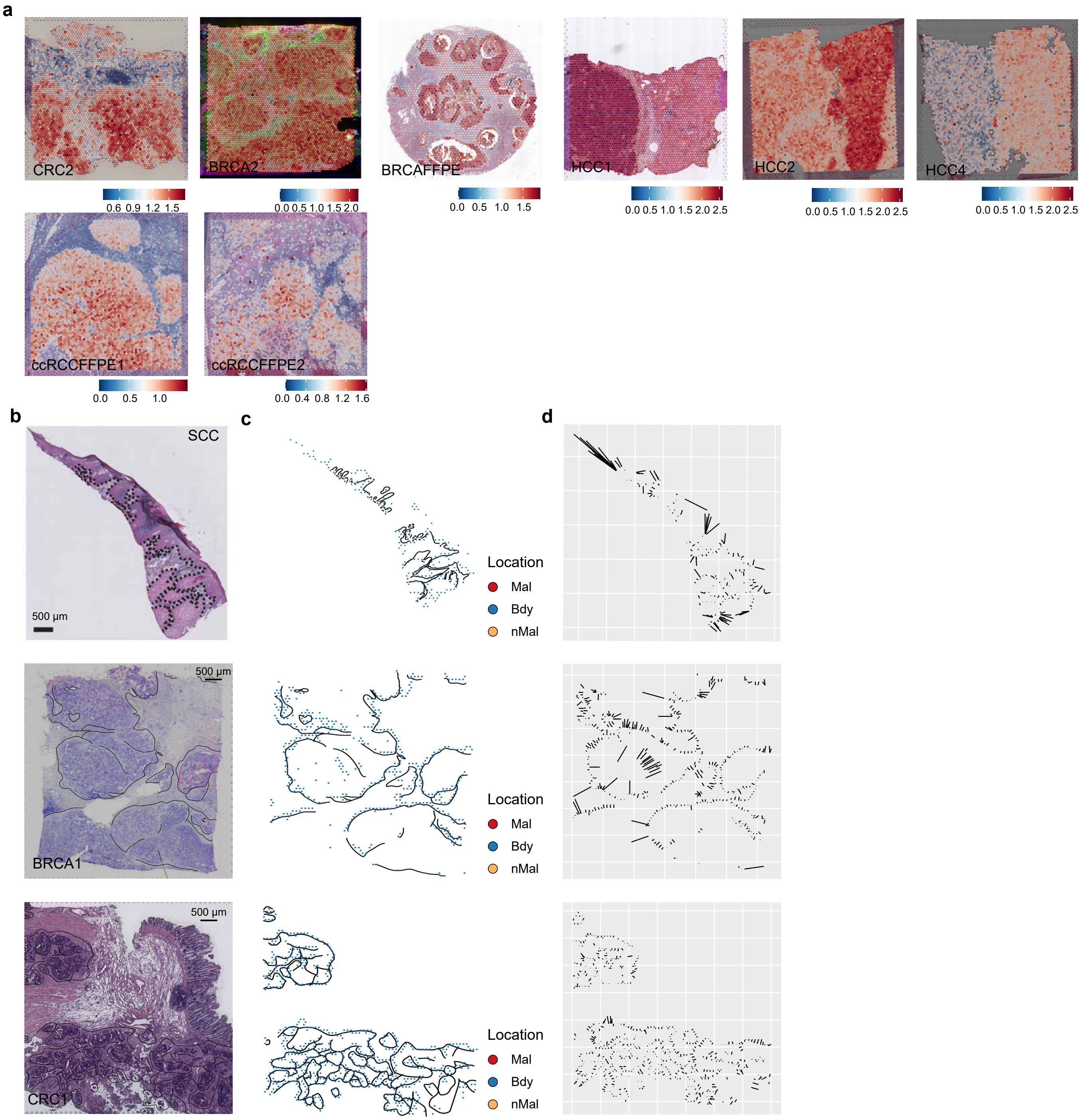

Supplementary Fig. 2 Performance of Cottrazm's boundary delineation function. Related to Figure 2. (**a**) Spatial feature plots of signature score of malignant spots in CRC (n = 1), BRCA (n = 1), FFPE BRCA (n = 1), HCC (n = 3), and ccRCC (n =2). (**b**) HE stained images with pathologist annotated tumor boundary of squamous cell carcinoma (SCC), breast cancer (BRCA), and colorectal cancer (CRC). Scale bar, 500 µm. (**c**) Tumor boundary annotated by pathologist (black) and boundary spots annotated by Cottrazm (Blue). (**d**) Line segments of the shortest distance from boundary spots annotated by Cottrazm to the pathologist's boundary.

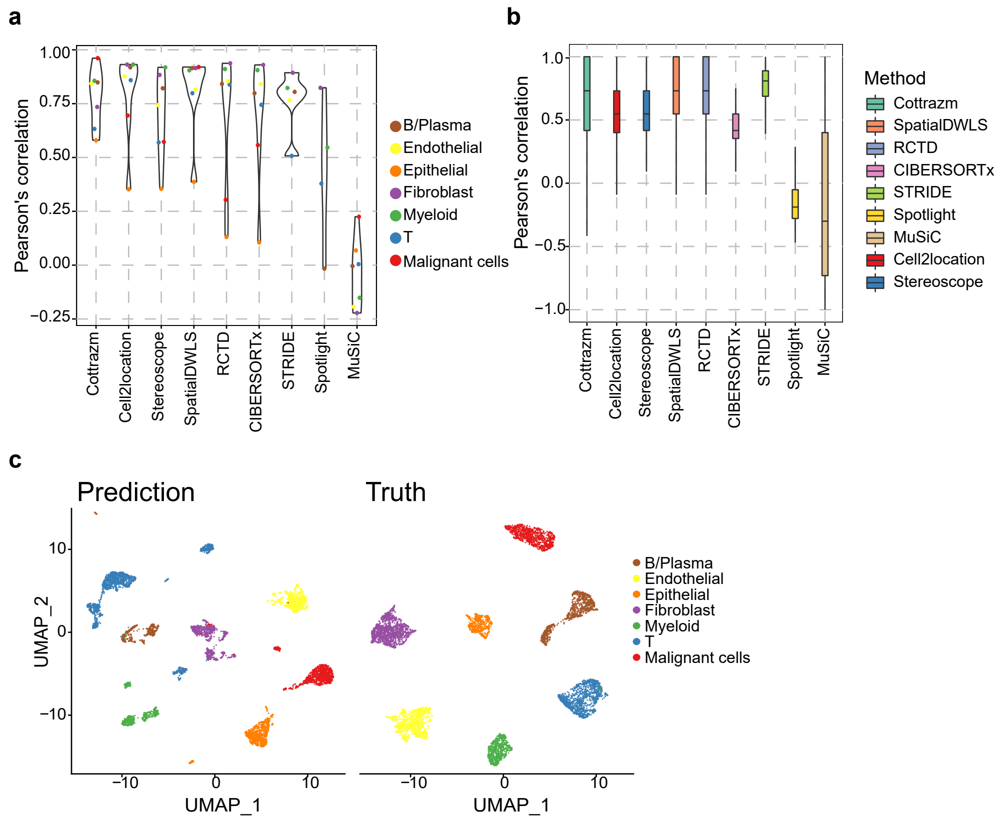

Supplementary Fig. 3 Performance of Cottrazm's deconvolution and reconstruction function using simulated data. Related to Figure 3. (**a**) A benchmark of the ability to distinguish different cell types across different deconvolution tools. Pearson's correlation was performed to evaluate the correlation between the predicted proportions and the ground truth for each cell type. (**b**) Benchmark of deconvolution tools' consistency of cell type distribution between the predicted proportions and the ground truth for each simulated spot (n = 2,700). The box plot reflects the overall distribution of Pearson's correlation calculated in each spot for each method. The boxes show the median ± 1 quartile, with the whiskers extending from the hinge to the smallest or largest value within 1.5 × the IQR from the box boundaries. (**c**) UMAP projections of cell types in the specific gene expression profiles (GEP) at sub-spot levels of prediction (left) and the ground truth (right). Source data are provided as Source Data Supplementary Figure 3c.

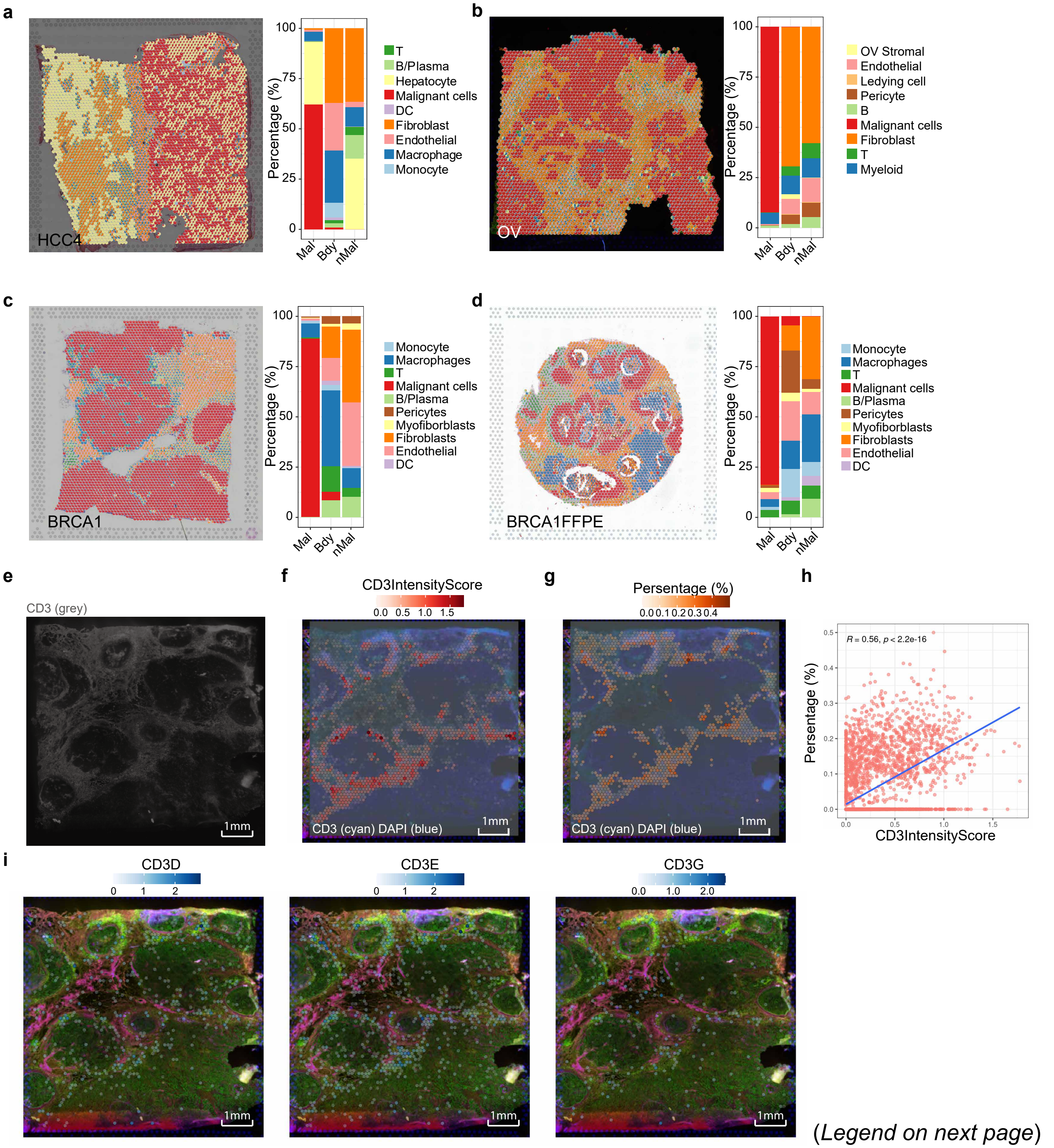

(*Legend on next page*)

Supplementary Fig. 4 Cottrazm accurately estimated the cell-type proportions in multiple cancer types. Related to Figure 4. Spatial scatter pie plots representing the proportions of the cell types predicted by Cottrazm (left) and bar plots representing the cell composition in malignant spots, boundary spots, and non-malignant spots (right) in HCC (**a**), OV (**b**), BRCA (**c**), and FFPE BRCA (**d**). (**e-h**) Comparation of the immunostaining, gene expression, deconvolution result of T cells in breast cancer. (**e**) Anti-CD3 antibody one-channel immunofluorescent imaging of the tissue section (N = 1 tissue section, n = 4,727 spots). (**f-g**) Spatial feature plots showed the CD3 intensity score (**f**) and the predicted proportion within each capture spot for T cells (**g**). Color indicates the percentage of cell type. (**h**) Scatter plot depicting the correlation between CD3 intensity score and predicted proportion of T cell infiltration. Pearson's (*R*) correlation coefficient of CD3 intensity score and predicted proportion of T cell infiltration. (**i**) Spatial feature plots showed the expression of *CD3D* (left), *CD3E* (middle), *CD3G* (right) of BRCA2 sample. Color indicates the gene expression. Scale bar, 1mm in **e-g** and **i**. Source data are provided as Source Data Supplementary Figure 4f-I.

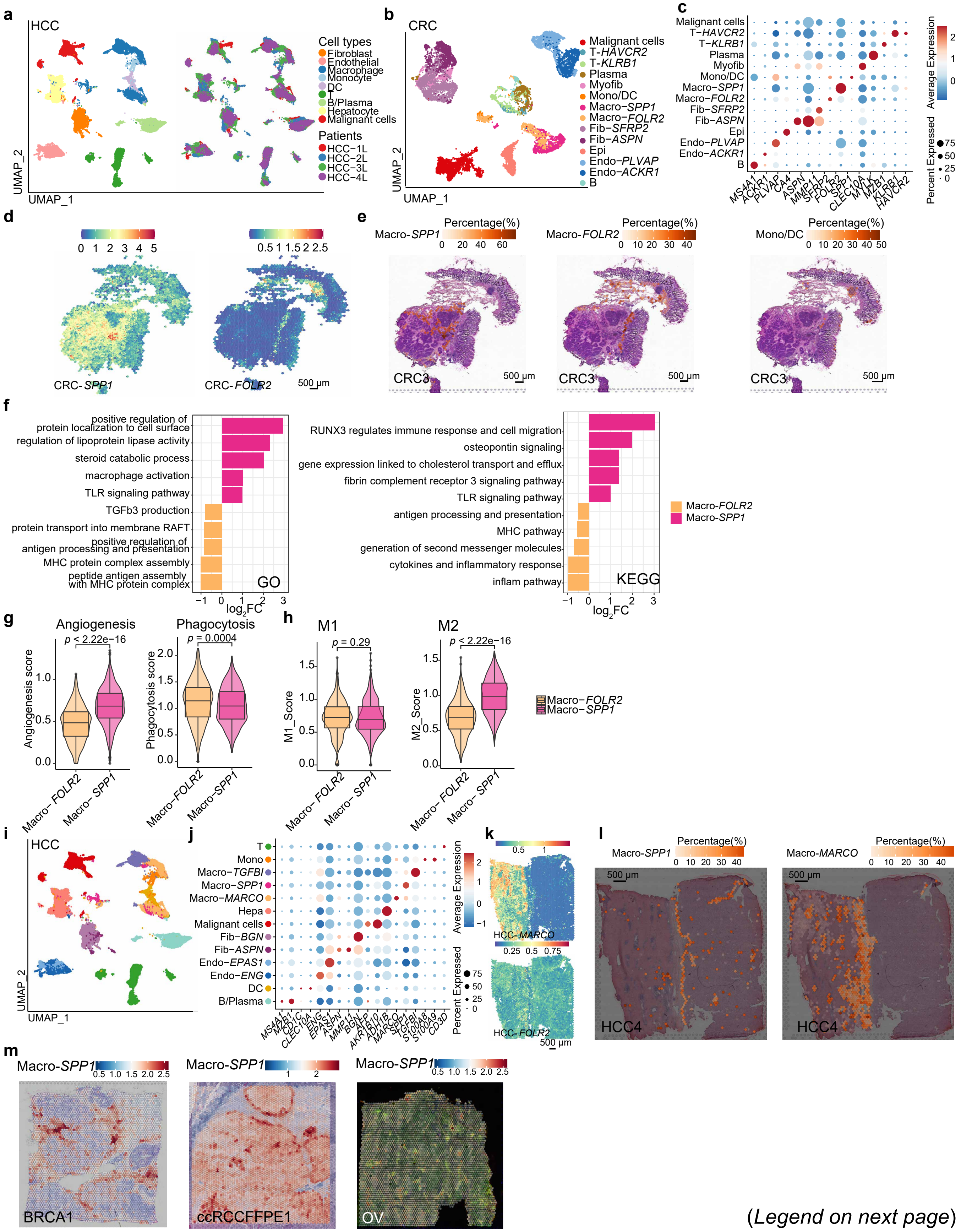

(*Legend on next page*)

Supplementary Fig. 5 Characterizing the heterogeneity of macrophage subtypes in spatial microenvironment. Related to Figure 4. (**a**) UMAP projections of sub-spots in three HCC ST dataset predicted by Cottrazm, each dot denotes one sub-spot; color represents cluster origin (left panel) or patient donors (right panel). (**b**) UMAP projections of sub-spots from gene expression profiles reconstructed by Cottrazm in CRC. (**c**) Dot plots showing average expression of known markers in indicated cell clusters in CRC. (d) Spatial feature plots showing the expression of *SPP1* and *FOLR2* in CRC. (**e**) Predicted proportion within each capture spot for Macro-*SPP1*, Macro-*FOLR2*, and Mono/DC in CRC. Color indicates the percentage of cell type. (**f**) Bar plots showing the differentially enriched Gene ontology (GO, left) and Kyoto Encyclopedia of Genes and Genomes (KEGG, right) terms of Macro-*SPP1* and Macro-*FLOR2*. (**g**) Box and violin plots showing the angiogenesis (left) and phagocytosis (right) scores of Macro-*SPP1* and Macro-*FOLR2* in reconstructed CRC data. (**h**) Box and violin plots showing the M1 (left) and M2 (right) scores of Macro-*SPP1* (n = 1,305) and Macro-*FOLR2* (n = 588) in reconstructed CRC data. The boxes show the median ± 1 quartile, with the whiskers extending from the hinge to the smallest or largest value within 1.5 × the IQR from the box boundaries. A two-sided Wilcoxon signed-rank test was used to assess statistical significance in **g** and **h**. (**i**) UMAP projections of sub-spots from gene expression profiles reconstructed by Cottrazm in HCC. (**j**) Dot plots showing average expression of known markers in indicated cell clusters in HCC. (**k**) Spatial feature plots showing the expression of *MARCO* and *FOLR2* in HCC. (**l**) Predicted proportion within each capture spot for Macro-*SPP1*, Macro-*MACRO*, and Mono/DC in HCC. (**m**) The signature score of Macro-*SPP1* in BRCA, ccRCC, and OV. Scale bar, 500 μm in **d-e** and **k-l**. Source data are provided as Source Data Supplementary Figure 5a-b, f, g-h.

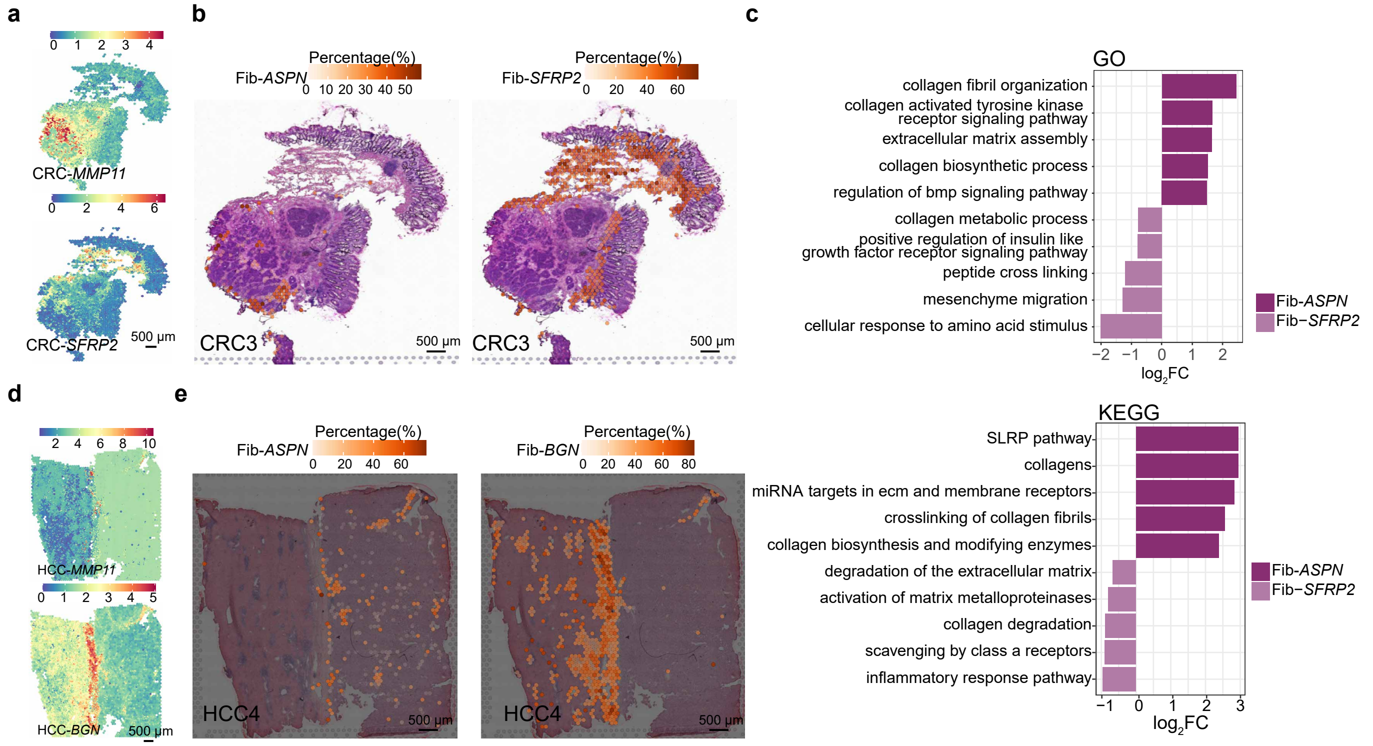

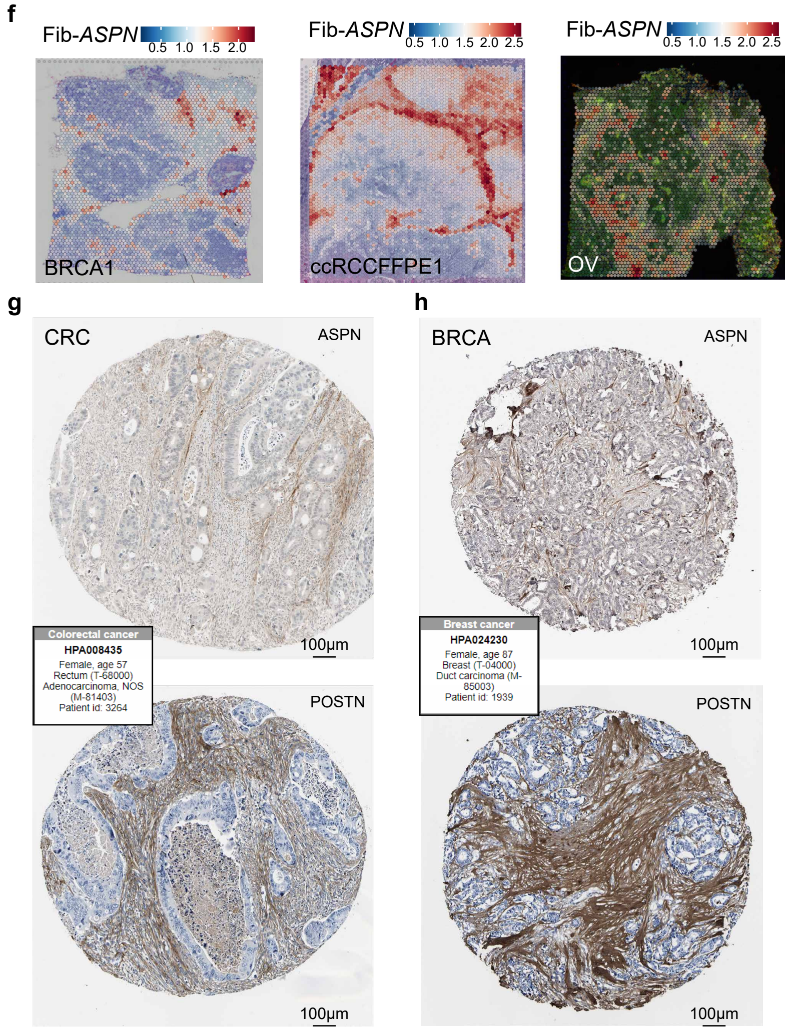

Supplementary Fig. 6 Characterizing the heterogeneity of macrophage subtypes in spatial microenvironment. Related to Figure 4. (**a**) Spatial feature plots showing the expression of *MMP11* and *SFRP2* in CRC. (**b**) Predicted proportion within each capture spot for Fib-*ASPN* and Fib-*SFRP2* in CRC. Color indicates the percentage of cell type. (**c**) Bar plots showing the differentially enriched GO (left) and KEGG (right) terms of Fib-*ASPN* and Fib-*SFRP2* in reconstructed CRC samples. (**d**) Spatial feature plots showing the expression of *MMP11* and *BGN* in HCC. (**e**) Predicted proportion within each capture spot for Fib-*ASPN* and Fib-*BGN* in HCC. Color indicates the percentage of cell type. (**f**) The signature score of Fib-*ASPN* in BRCA, ccRCC, and OV. Scale bar, 500 µm in **a-b** and **d-e**. (**g-h**) Immunostaining of ASPN (upper) and POSTN (bottom) in CRC and BRCA samples obtained from human protein atlas.Scale bar, 100 µm in **g-h**. Source data are provided as Source Data Supplementary Figure 6c.

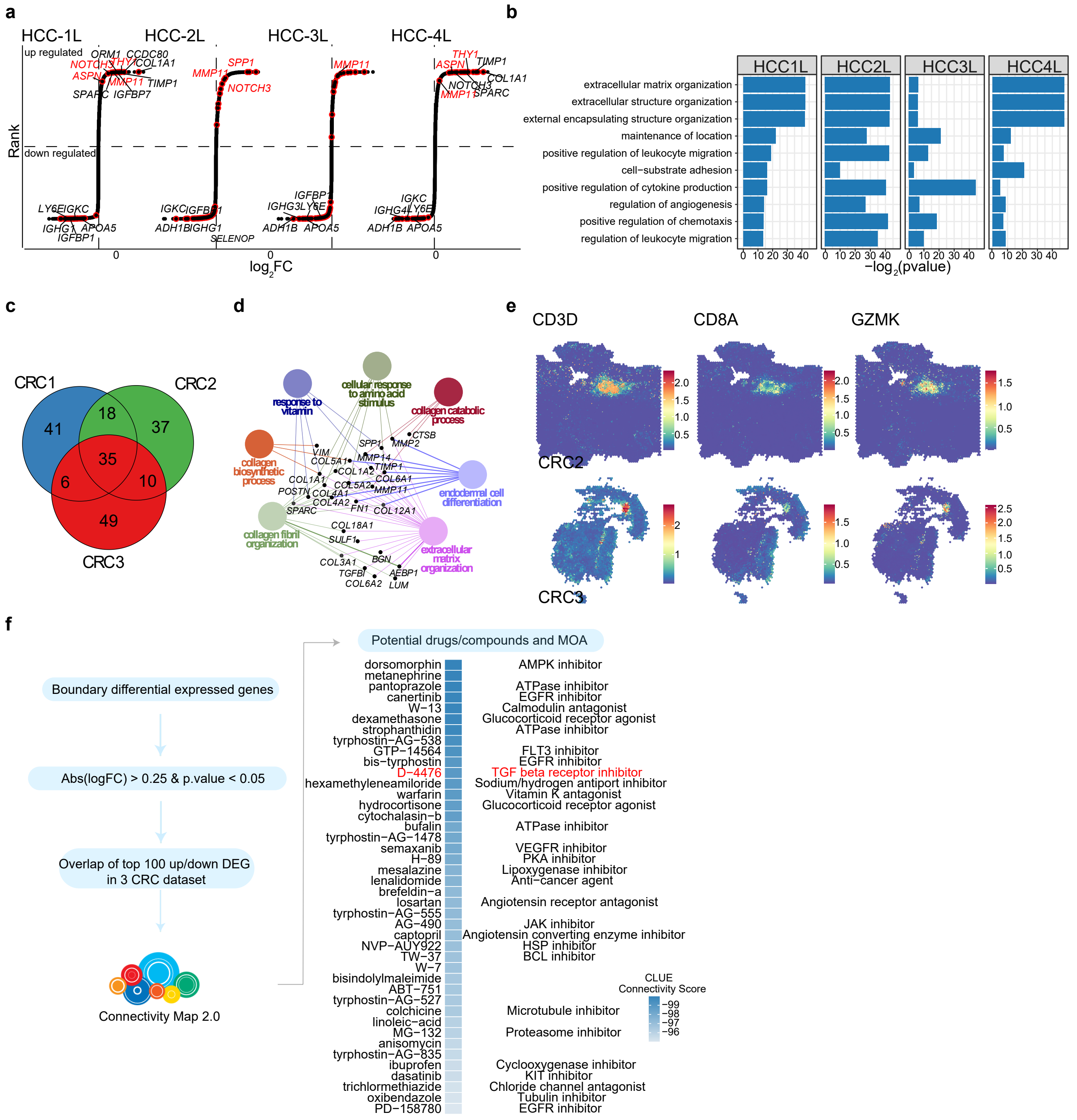

Supplementary Fig. 7 Characterization of the tumor boundary microenvironment. Related to Figure 5. (**a**) Rank-ordered plot showing log2 fold change between gene expression in tumor boundary and other regions in four HCC ST samples. (**b**) GO terms of genes significantly enriched in tumor boundary four HCC ST samples. The statistical analysis was performed by two-sided Fisher's test. (**c**) Venn diagram showing the intersection of top 100 differentially expressed genes in the boundary spots of 3 CRC patients. (**d**) Protein–protein interaction network of the query signature in overlapping genes of the top 100 specifically expressed genes in the tumor boundary of the three CRC ST samples as the signature genes. The protein–protein interactions were obtained from the STRING database. The width of the line indicates the edge confidence. Several significantly enriched biological processes are highlighted by different colors. (**e**) Spatial feature plot showing the expression of *CD3D*, *CD8A*, and *GZMK* in CRC2 and CRC3. (**f**) Drug prediction in the tumor boundary. The overlapping genes of the top 100 upregulated and 100 down-regulated genes in the tumor boundary of the three CRC ST samples were used as the query signature to match the reference profiles of perturbagens in connectivity map (CMAP) to calculate connectivity scores. Perturbagens are sorted by connectivity score in increasing order, and the top perturbagens are predicted as candidate drugs. DEG, differentially expressed genes; MOA, mechanism of action. Source data are provided as Source Data Supplementary Figure 7a-b, f.

Supplementary Table 1. Summary of spatial transcriptomics data in this study

Sample	Tissue	Total spots	Median UMIs/spot	Median genes/spot	Median mitochondrial genes/spot (%)
CRC1	Frozen	4457	7,518	3,083	11.23
CRC2	Frozen	3892	4,830	2,051	14.36
CRC3	Frozen	1657	16,868	4,379	11.35
BRCA1	Frozen	3798	20,762	6,026	3.50
BRCA2	Frozen	4727	6,314	2,694	6.20
BRCAFFPE	FFPE	2518	14,442	5,244	\
HCC1	Frozen	2791	16,407	4,017	1.25
HCC2	Frozen	4672	6,604	2,876	1.40
HCC3	Frozen	4758	9,348	3,044	7.70
HCC4	Frozen	4113	3,861	12,919	5.32
ICC	Frozen	4654	4,648	14,791	3.17
OV	Frozen	3493	8,095	3,464	7.56
ccRCC	Frozen	2007			3.35
ccRCCFFPE1	FFPE	4975			\
ccRCCFFPE2	FFPE	4948			\
SCC	Frozen	744	16947	3508	2.61

Supplementary Table 2. Tumor specific signature in cancers

CRC	BRCA	HCC	OV	ICC	ccRCC
ALCAM	*YBX1*	*AFP*	*EPCAM*	*KRT19*	*CA9*
CD24	*ENO1*	*FABP1*	*WFDC2*	*KRT7*	*ANGPTL4*
LGR5	*ERBB2*	*SERPINA1*	*TFPI2*	*ANXA4*	*NDUFA4L2*
EPCAM	*KRT18*	*APOA2*	*TACSTD2*	*AKR1C2*	*NNMT*
ALDH1A1	*GATA3*	*NTS*	*SLPI*	*SNCG*	*FGG*
CDCP1	*FOXA1*	*NUPR1*	*CLDN4*	*FABP5*	*HILPDA*
DPP4	*ESR1*	*GPC3*	*PAX8*	*FDCSP*	*SLC17A3*
MME	*CD24*	*AKR1B10*	*MYC*	*FXYD3*	*CD40*
DPEP1	*EPCAM*	*SPINK1*	*MMP7*	*EPCAM*	*CD70*
KRT8	*CCND1*	*PROM1*	*IFITM3*	*ALDH1A1*	*HHLA2*
FABP1	*CDH1*	*CD24*	*FTH1*	*CD24*	
ASCL2	*KRT7*		*WT1*	*FCGBP*	
PLS3	*KRT18*		*CD24*	*KRT81*	
OLFM4	*KRT19*			*CALB1*	
BMI1	*AR*			*CST6*	
DPEP1	*FOXA1*			*EPCAM*	

Abbreviation: CRC, colorectal cancer; BRCA, breast cancers, HCC, hepatocellular carcinoma; OV, ovarian cancer; ICC, intrahepatic cholangiocarcinoma; ccRCC, clear cell renal cell carcinoma.
